# H_2_S- and NO-Signaling Pathways in Alzheimer's Amyloid Vasculopathy: Synergism or Antagonism?

**DOI:** 10.3389/fphys.2015.00361

**Published:** 2015-12-11

**Authors:** Alla B. Salmina, Yulia K. Komleva, István A. Szijártó, Yana V. Gorina, Olga L. Lopatina, Galina E. Gertsog, Milos R. Filipovic, Maik Gollasch

**Affiliations:** ^1^Department of Biochemistry, Medical, Pharmaceutical and Toxicological Chemistry, Krasnoyarsk State Medical University named after Prof. V.F. Voino-YasenetskyKrasnoyarsk, Russia; ^2^Experimental and Clinical Research Center, Charité - University Medicine Berlin and the Max Delbrück Center for Molecular MedicineBerlin, Germany; ^3^Department of Chemistry and Pharmacy, Friedrich-Alexander-University of Erlangen-NürnbergErlangen, Germany

**Keywords:** H_2_S, vascular dysfunction, Alzheimer disease (AD), NO, KCNQ channels, obesity

## Abstract

Alzheimer's type of neurodegeneration dramatically affects H_2_S and NO synthesis and interactions in the brain, which results in dysregulated vasomotor function, brain tissue hypoperfusion and hypoxia, development of perivascular inflammation, promotion of Aβ deposition, and impairment of neurogenesis/angiogenesis. H_2_S- and NO-signaling pathways have been described to offer protection against Alzheimer's amyloid vasculopathy and neurodegeneration. This review describes recent developments of the increasing relevance of H_2_S and NO in Alzheimer's disease (AD). More studies are however needed to fully determine their potential use as therapeutic targets in Alzheimer's and other forms of vascular dementia.

## Cerebrovascular dysfunction in AD

Due to the increasing prevalence and high severity, Alzheimer's disease (AD) is one of today's major health challenges. AD typically manifests after the age of 60. As the population ages, this disease impacts a greater percentage of the world-wide population. AD is a progressive neurodegenerative disorder characterized by deposition of amyloid-beta (Aβ) in the brain tissue that leads to cognitive, memory, and behavioral impairments. Several hypotheses were proposed to explain the pathogenesis of AD: Aβ toxicity, cholinergic dysfunction, abnormal phosphorylation of *tau* protein, development of oxidative stress due to action of endogenous or exogenous pro-oxidants, impairment of neurogenesis, excessive cell death, neuroinflammation etc. (Salmina, 2009).

The following strategies have been proposed for the pharmacotherapy of AD: (1) prevention of accumulation of aberrant proteins (suppression of Àβ production or activation of Àβ clearance); (2) modulation of neuroplasticity; (3) suppression of neuroinflammation; (4) stimulation of neurogenesis and other brain repair mechanisms. However, treatments aimed at reducing Aβ deposits showed no or little success, therefore, alternative approaches appear to be more prospective. In this context, current prevention and treatment strategies are increasingly focused on AD associated vasculopathy.

Cerebrovascular dysfunction is not only a marker of ischemic brain pathology, but also of neurodegenerative disorders such as AD. The vascular hypothesis of AD suggests that neurodegenerative pathology begins with cerebral hypoperfusion and cerebral microvascular abnormalities associated with extensive Aβ deposition and blood-brain barrier disruption. Contribution of cerebrovascular dysfunction to the pathogenesis of AD is evident not only in humans (Saito et al., 2015) but also in experimental models of AD (Han et al., 2015). It is associated with prominent oxidative stress, Aβ-impaired cerebral circulation, and alterations in the neurovascular unit or blood-brain barrier (Han et al., 2008; Bell and Zlokovic, 2009).

Deposition of Aβ is an important mechanism of cerebrovascular dysfunction in AD, and cerebral amyloid angiopathy facilitates progression of AD and cognitive impairment. Cerebral amyloid angiopathy in AD is caused by the accumulation of Aβ in small-sized and medium-sized blood vessels, mostly in arteries (Biffi and Greenberg, 2011). In severe angiopathy, amyloid deposits replace degenerating vessel smooth muscle cells, thus resulting in microhemorrhages, ischemic lesions, and encephalopathies (Yamada, 2015). Accumulation of Aβ leads to extensive neoangiogenesis and hypervascularity associated with abnormal blood-brain barrier leakiness in AD (Biron et al., 2011), but the vessels have smaller diameter suggesting vasomotor dysfunction and/or vascular remodeling in AD (Burke et al., 2014). Mechanisms of cerebral amyloid angiopathy in AD include insufficiency of perivascular drainage of Aβ leading to its accumulation in the vessel wall; development of perivascular inflammation and microhemorrhages, vascular oxidative stress (Park et al., 2011; Hawkes et al., 2014; Boncoraglio et al., 2015). We also found that Aβ peptides elicit a signal transduction pathway in vascular cells, induced by α1-adrenergic receptor activation (Haase et al., 2013). However, it is not clear which molecular events and amyloid toxic action affect the time-course of neurodegeneration. Nevertheless, it is clear that there is a strong correlation between the prevalence of cerebral amyloid angiopathy and age. As such, up to 40% of the elderly population without clinical manifestations of AD demonstrate features of cerebral amyloid angiopathy, and up to 80% of people suffering from AD exhibit signs of cerebral amyloid angiopathy (Jellinger, 2002). Deciphering the molecular mechanisms of cerebral amyloid angiopathy would be beneficial for development of novel therapeutic and diagnostic strategies.

Endothelial cells in the cerebral vasculature may contribute to the formation of amyloid deposits surrounding the cerebral blood vessels. Several recent studies have highlighted that endothelial cells might be the target for the toxic action of heavily aggregated proteins, glia-derived cytokines, and stimuli inducing oxidative and metabolic stress in AD brains (Salmina et al., 2010). Cerebral endothelial cells are in the close connection with pericytes, astrocytes, and neurons in the neurovascular unit, therefore altered paracrine and autocrine interactions of these cells might be critically involved in the development of microvascular abnormalities in AD.

## Gaseous transmitters in the brain: general characteristics

**Figure 1:**
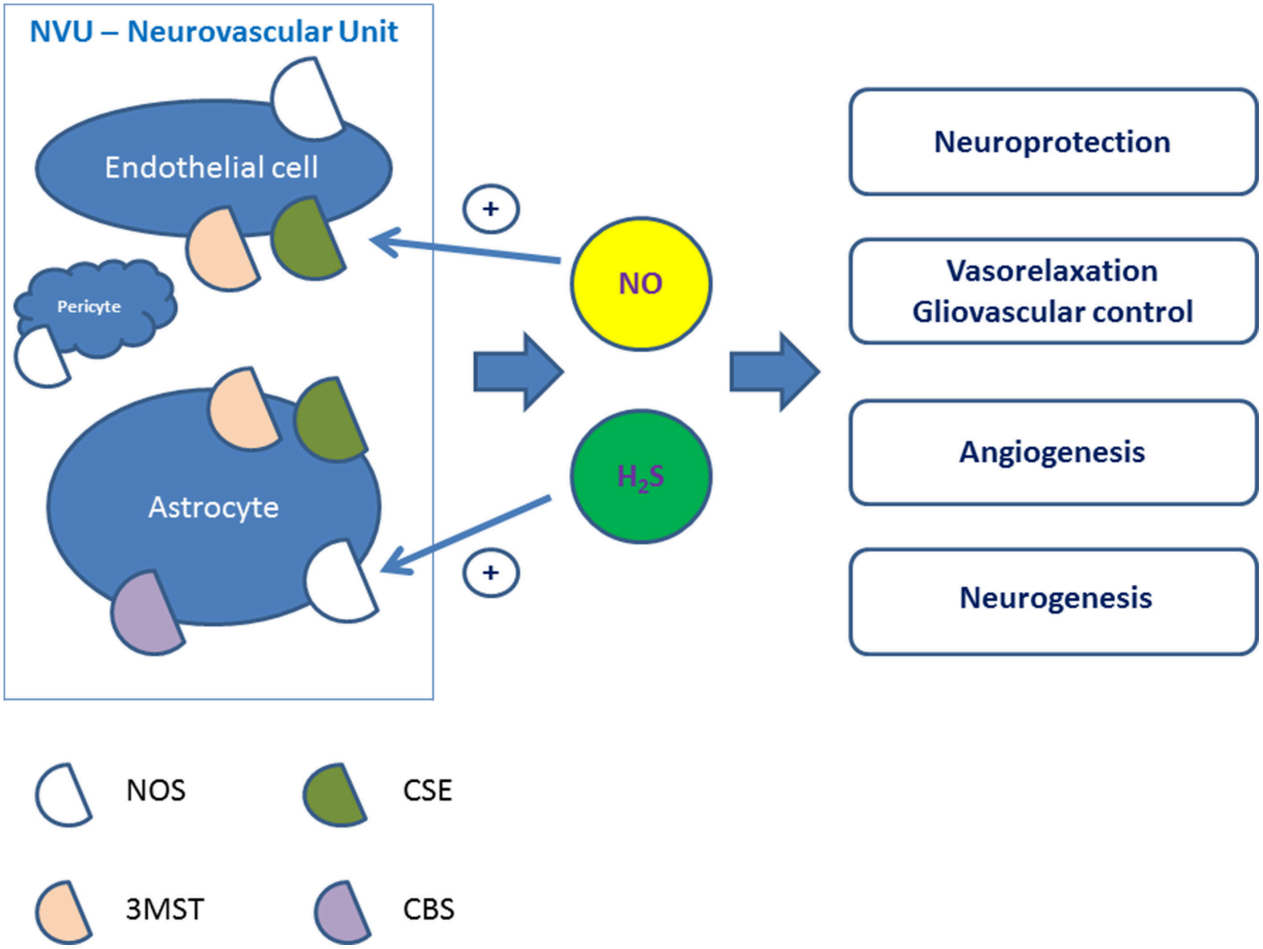
**Putative physiological effects of NO and H_2_S produced in the cells of the neurovascular unit**. NVU, neurovascular unit; NOS, nitric oxide synthase; MST, 3-mercaptopyruvate sulfurtransferase; CSE, cystathionine-γ-lyase; CBS, cystathionine-β-synthase.

Production of gaseous transmitters by mammalian cells has attract much attention in past few years. Hydrogen sulfide (H_2_S) is now considered the third gaseous transmitter besides nitric oxide (NO) and carbon monoxide (CO) to contribute to the regulation of cardiac function, systemic and pulmonary blood pressure and vasomotor activity, control inflammation, and angiogenesis (Köhn et al., 2012; Lo Faro et al., 2014). Being produced by various cell types, these gaseous transmitters can easily penetrate the plasma membrane thus inducing wide spectrum of signaling cascades in the target cells. In brain, H_2_S, CO, and NO are released by astroglial cells, neurons, or endothelial cells (Figure 1), and can functionally interact.

**Figure 2:**
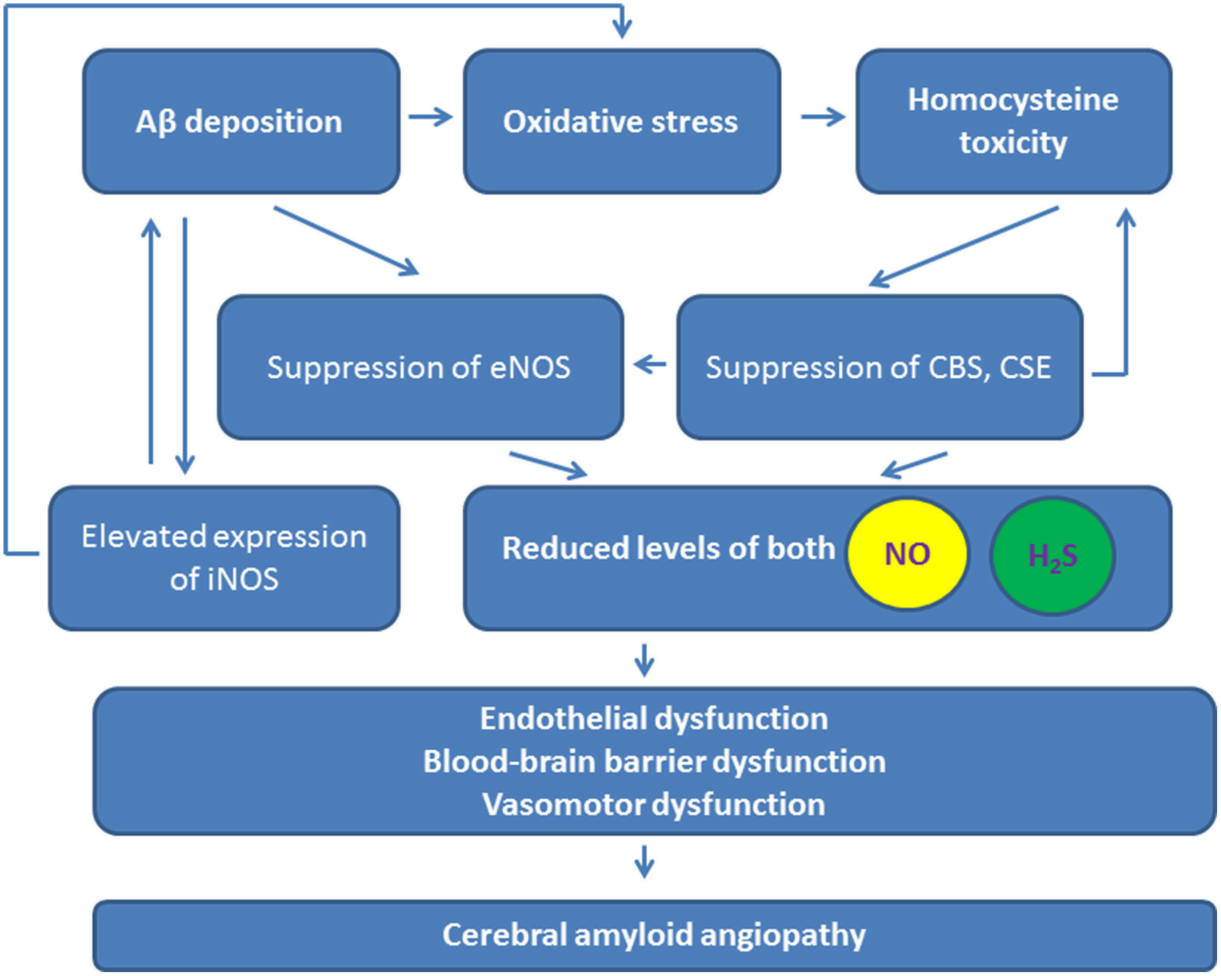
**Contribution of NO and H_2_S to the pathogenesis of cerebral amyloid angiopathy**. Purple arrows, reduced levels and actions of NO and H_2_S. iNOS, inducible nitric oxide synthase; eNOS, endothelial nitric oxide synthase; CSE, cystathionine-γ-lyase; CBS, cystathionine-β-synthase.

The action of NO in the brain is extensively studied. NO is now known as a potent vasorelaxant, neurotransmitter, pro-inflammatory, and pro-oxidant molecule (Guix et al., 2005; Kovac et al., 2011; Tabatabaei and Girouard, 2014), even there are some data on antioxidant activity of NO (Hummel et al., 2006). Coordinated expression and activity of nitric oxide synthase (NOS), as either constitutive or inducible isoforms, in endothelial and neuronal cells regulates local NO release to contribute to local neurovascular and metabolic coupling, and neuronal excitability. Molecular targets of NO are cysteine and tyrosine residues in cell proteins that can be nitrosylated or nitrated, respectively (Hess et al., 2005). Although the neurotoxic *vs.* neuroprotective activity of NO is still a matter of debate, and even dose-dependent effects should be considered (Tripathy et al., 2015), the majority of the data suggests that dysregulated NO production facilitates neurodegeneration (Figure 2; Jullienne and Badaut, 2013).

Almost similar activities can be attributed to CO produced by heme oxygenase. CO acts as a modulator of cerebral vasomotor function (Parfenova et al., 2012b), but in the contrast to NO which can easily react with superoxide anion to produce peroxynitrite (i.e., in proinflammatory conditions or in reperfusion) with the ultimate pro-oxidant activity, CO exerts anti-oxidant properties (Parfenova et al., 2012a). Molecular targets of CO are potassium (BK_Ca_) channels, guanylyl cyclase, NADPH oxidase, and the heme-containing components of the mitochondria respiratory chain (Parfenova and Leffler, 2008). In general, CO is considered to be a cytoprotective mediator in the brain, and elevation of its level may improve cerebrovascular outcome of brain injury (Liu et al., 2015).

Endogenous H_2_S acts as a potent regulator of various biological processes mainly related to vasomotor function (Figure 2). H_2_S regulates intracellular calcium concentrations *via* L-type calcium channels, T-type calcium channels, sodium/calcium exchangers, transient receptor potential channels, β-adrenergic receptors, and N-methyl-D-aspartate receptors (NMDA) in various cells (Zhang et al., 2015).

Exogenous H_2_S can produce relaxation of a number of non-cerebral systemic arteries by mechanisms involving opening of adenosine 5′-triphosphate (ATP)-sensitive potassium (K_ATP_) channels, intermediate (IK) and small conductance potassium channels (SK), KCNQ-type voltage-gated potassium (K_v7.x_) channels, regulating the Cl^−^/${\text{HCO}}_{3}^{-}$ transporter, decreasing adenosine triphosphate levels, and/or release of endogenous vasodilatory prostanoids (Köhn et al., 2012). Of note, exogenous H_2_S can produce vasoconstrictions at low concentrations (< 100 μM) in some vessels, probably *via* inhibition of cAMP/protein kinase A (PKA) pathway in smooth muscle cells and/or interaction with the endothelial nitric oxide synthase/nitric oxide (eNOS/NO) pathway (Kubo et al., 2007a).

The biological effects of H_2_S in the central nervous system and cerebral circulation are less clear. Cerebral vessels express H_2_S-generating enzymes (Chertok and Kotsyuba, 2012), thus, cerebral endothelial cells may use H_2_S to induce cerebrovascular relaxation to increase local blood flow. H_2_S produced by astrocytes or endothelial cells within the neurovascular unit may mediate gliovascular control adjusting local blood perfusion to the actual needs in the activated brain area (Figure 1). Biological effects of H_2_S include modulation of neuronal excitability, regulation of vessel tone, anti-oxidant and anti-inflammatory activity, regulation of vascular tone, angiogenesis, and blood-brain barrier permeability (Geng et al., 2015; Kimura, 2015). In general, H_2_S demonstrates neuroprotective properties (Zhang and Bian, 2014), but the sensitivity of the cells to H_2_S depends on developmental status of the cells: differentiated cell have greater sensitivity to H_2_S compared to progenitor cells (Tsugane et al., 2007).

There is evidence that microvascular function is not controlled by the activity of these three gaseous transmitters independently by themselves, but rather by a complex interaction between NO, CO, and/or H_2_S gases, which enables a complex pattern of hemodynamic microvascular control in the brain (Dyson et al., 2014) and can affect the function of neuronal cells (Pong and Eldred, 2009). As an example, nitrosothiols are organic compounds or functional groups containing a nitrosogroup attached to the sulfur atom of a thiol. S-Nitrosated proteins (SNOs) serve to transmit nitric oxide (NO) bioactivity *in vivo*; their reaction with H_2_S, however, results in the formation of HSNO which can promote further trans-nitrosation of specific protein targets or give NO and nitroxyl (HNO), both of which could have biological activity aimed to promote smooth muscle cell relaxation (Filipovic et al., 2012). Oxidation of H_2_S could lead to formation of polysulfides, which are very reactive with cell tiols (Greiner et al., 2013; Wedmann et al., 2014). TRPA1 channels are proposed to represent potential targets for the stimulatory effects of polysulfides in the cells (Hatakeyama et al., 2015), and these channels are importantly also affected by lipid peroxidation metabolites and glycolysis by-product methylglyoxal (Eberhardt et al., 2012; Sullivan et al., 2015). Activation of TRPA1 channels enables Ca^2+^ signal-effector coupling at discrete sites along the endothelium to evoke graded cerebral artery vasodilation (Qian et al., 2013).

## H_2_S-generating machinery in brain cells

H_2_S is produced from cysteine due to the activity of various enzymes (Figure 1). Within the neurovascular unit, cystine as a cysteine precursor is taken up by astrocytes and then cysteine is released from astroglial cells for neuronal and endothelial needs (Guebel and Torres, 2004). Import of l-cystine into astrocytes and corresponding efflux of l-glutamate is provided by the x(c)—antiporter whose expression is elevated in neuroinflammation or brain hypoxia (Jackman et al., 2010, 2012). Cysteine is further converted into H_2_S or taurine (with anti-oxidant and anti-inflammatory activities), and is a mainly used for glutathione synthesis.

The endogenous levels of H_2_S in the brain and various organs were recently re-evaluated and found to be much lower than previously estimated (Ishigami et al., 2009; Wintner et al., 2010). Nevertheless, H_2_S is generated by two major enzymes, namely cystathionine β-synthase (CBS) and cystathionine γ-lyase (CSE) using vitamin B_6_ as cofactor. Some authors believe that CSE plays a major role in generating H_2_S in cardiovascular system, while CBS is responsible for H_2_S production in the brain (Wang et al., 2014), i.e., by activated astrocytes due to shift in intracellular pH required for release of H_2_S from bound sulfur (Ishigami et al., 2009). CSE, but not CBS, was detected in cerebral microvessels, whereas CBS was detected in brain parenchyma (Leffler et al., 2011).

CBS and CSE may also use homocysteine for H_2_S synthesis (Wang, 2012): initial conversion of homocysteine to cyctathionine followed by conversion to cysteine. When H_2_S is synthesized from cysteine due to activity of CBS or CSE/3MST, l-serine or pyruvate are the side products, respectively (Olson et al., 2013).

Brain transsulfuration pathway involving cysteine, homocysteine, and cystathionine generates H_2_S and the atypical amino acid lanthionine (Hensley and Denton, 2015). The latter regulates brain cells autophagy (Harris-White et al., 2015), neuritis outgrowth (Hubbard et al., 2013), and is currently discussed as a candidate for correction of neurological alterations seen in neurodegeneration (Hensley and Denton, 2015).

Increased expression of transsulfuration pathway enzyme CSE is caused by dietary restriction, particularly, by shortage in the sulfur amino acids consumption. As a result, H_2_S production is elevated and its cytoprotective properties become more evident. It was proposed that dietary restriction-mediated positive effect on longevity might have a relation to permanent H_2_S-mediated protection from various pathogenic stimuli (Hine et al., 2015).

Since CSE expression was not confirmed in brain cells, CBS is considered to be the major H_2_S-producing enzyme in the brain. In agreement, astroglial cells express CBS at high level (Wang, 2012). However, H_2_S was identified in the brain of CBS-knockout mice. This led to the identification of a third H_2_S-generating pathway, which is regulated by 3-mercaptopyruvate sulfurtransferase (3MST) along with cysteine aminotransferase (CAT; Tanizawa, 2011) in the presence of thioredoxin. Although expression of CBS is mainly attributed to astrocytes (Enokido et al., 2005) and microglia (Du et al., 2014), 3MST is mainly expressed in neurons and endothelial cells (Figure 1). Of note, down-regulation of 3MST expression was detected in astroglial cells after stroke (Zhao et al., 2013). In general, astrocytic production of H_2_S is almost 10 times higher compared to H_2_S production in microglial cells (Wang et al., 2014). Endothelial cells expressing 3MST represent also a source for H_2_S production from cysteine and alpha-ketoglutarate (Shibuya et al., 2009); noteworthy, cerebral endothelial cells may use H_2_S to induce smooth muscle cell relaxation to increase local blood flow. Therefore, H_2_S produced by astrocytes or endothelial cells within the neurovascular unit may mediate gliovascular control adjusting local blood perfusion to the actual needs in the activated brain area (Figure 1). Nevertheless, it is still a matter of debate, which enzyme(s) is (are) responsible for brain injury-associated alterations in endogenous H_2_S production (Zhao et al., 2013). Recently, it was found that target inhibition of different H_2_S-generating enzymes can be achieved in experimental conditions by pharmacological approach, i.e., with DL-propargylglycine, which is an inhibitor for CSE, aspartate which is an inhibitor for 3MST, or O-(carboxymethyl)hydroxylamine hemihydrochloride, which is an inhibitor for CBS (Jiang et al., 2015). This approach revealed pivotal roles of 3MST and CSE in ischemia-reperfusion-associated blood-brain barrier alterations.

It is generally accepted that H_2_S and NO interact with each other at the level of expression of the enzymes producing these molecules where mutual down-regulation effect is demonstrated (Rong-na et al., 2011; Figure 2). In macrophages, both NO and H_2_S irreversibly suppress NOS activity (Heine et al., 2015), thus providing negative feedback control. In contrast, there are some reports on mutually stimulatory activity of these two gases on their production (Kolluru et al., 2013). Therefore, H_2_S may stimulate NO production in endothelial cells to promote neoangiogenesis (Altaany et al., 2013) (Figure 1). However, some authors report that eNOS can be directly inhibited by H_2_S (Kubo et al., 2007b), and this effect can underlie neuroprotective effects of H_2_S in neonatal brain hypoxia (Wang et al., 2013b). Since post-ischemic upregulation of eNOS triggers cerebral angiogenesis accompanied by increased BBB permeability, ischemia/hypoxia-induced vascular plasticity might be regulated by the altering production of NO and H_2_S in different phases of ischemia and reperfusion in order to provide adequate proangiogenic microenvironment and to prevent dramatic alterations in the BBB structural integrity. Thus, H_2_S and NO produced within the neurovascular unit may attenuate vasogenic brain edema formation and protect the brain by enhancing cerebral blood flow and neoangiogenesis.

H_2_S can produce vasorelaxation due to inhibition of RhoA-dependent signaling cascades in SMC (Nalli et al., 2015). The same is true for NO action (Sawada et al., 2001). Taking into consideration that inhibition of RhoA downstream molecular machinery (i.e., Rho-associated coiled-coil forming protein kinases) leads to the upregulation and activation of eNOS (Noma et al., 2012), one can propose that H_2_S may also exert NOS-stimulatory effects under (patho)physiological conditions (Kram et al., 2013). However, this effect has not been demonstrated in brain cells so far. Similarly, H_2_S-induced up-regulation of heme oxygenase expression has been observed in various non-cerebral tissues (Zhang et al., 2013; D'Araio et al., 2014; Wang et al., 2015), however, evidence for such an effect in brain cells is missing.

More importantly there is increasing body of evidence that H_2_S can react directly with NO (Yong et al., 2010; Filipovic et al., 2013; Eberhardt et al., 2014; Dux et al., 2015). Nitroxyl (HNO), as a one-electron reduced sibling of nitric oxide, has been proposed as a main mediator of this direct reaction between NO and H_2_S. Our research team has shown that nitroxyl, formed in the reaction of H_2_S and NO, can activate TRPA1 channels by oxidizing critical cysteine residues which leads to Ca^2+^ influx and subsequent calcitonin gene-related peptide (CGRP) release from sensory nerve ending. This could contribute to systemic blood pressure regulation as well as regulation of cerebral blood flow (Eberhardt et al., 2014). The importance of this H_2_S+NO/HNO/TRPA1/CGRP signaling cascade has been recently established in the context of meningeal blood flow and pathology of migraines (Dux et al., 2015).

Thus, the cells of the neurovascular unit (endothelial cells, astrocytes) produce NO and H_2_S whose biological effects support intercellular communications, gliovascular control, cell proliferation and development (Figure 1). Compromised production or action of NO and H_2_S may be associated with the development of various neurodegenerative and cerebrovascular disorders.

## H_2_S and NO in the alzheimer's type of angiopathy

### Vascular Aβ deposition

Under normal conditions, NO protects endothelial cells and adjusts cerebrovascular function to the actual blood flow needs in active brain regions. NOS activity in endothelial cells prevents APP overexpression and Aβ synthesis (Katusic and Austin, 2014). In AD, Aβ impairs endothelial function due to inhibition of eNOS activity caused by alterations in intracellular calcium homeostasis and protein phosphorylation pattern (Gentile et al., 2004). Deposition of Aβ, thickening and hyalinization of the media of small and medium-size vessels, and apoptosis of endothelial and smooth muscle cells correlate with reduced NOS expression in cerebral vessels (de la Monte et al., 2000). Deficiency of NOS results in increased Aβ production and neuroinflammation development (Austin et al., 2013). These alterations are accompanied with development of oxidative stress (Lamoke et al., 2015). Oxidative stress is a key contributor to development of cerebral amyloid angiopathy, vessel constriction, and cerebral amyloid angiopathy-related microhemorrhages (Han et al., 2015). Thus, permanent inhibition of eNOS in endothelial cells results in vascular dysfunction and impairment of cerebral microcirculation (Lin et al., 2014), and leads to further progression in Aβ tissue deposition. Endothelial eNOS affects transport of Aβ through the blood-brain barrier, therefore, dysregulation of eNOS activity or expression would lead to promotion of amyloid deposition in the brain tissue as it was previously shown in Provias and Jeynes (2014) (Figure 2).

H_2_S can also inhibit Aβ production in the cells due to suppression of γ-secretase activity (Nagpure and Bian, 2014) and an inhibit Aβ deposition, most likely, due to suppression of fibril formation (Rosario-Alomar et al., 2015). However, eNOS activity can be stimulated by H_2_S in endothelial cells (Chen et al., 2014) via calcium ions release from intracellular stores (Kida et al., 2013). Thus, cerebral amyloid angiopathy-associated changes in eNOS expression and activity might be linked to dysregulated production of H_2_S. Indeed, in AD, CBS activity, and H_2_S production are reduced in the brain (Eto et al., 2002), and plasma H_2_S levels are negatively correlated with the severity of AD (Giuliani et al., 2013), thus, neuroprotective and angioprotective properties of H_2_S are reduced (Figure 2). Therefore, it is not surprising that restoration of H_2_S levels can exhibit beneficial effects in AD (Fan et al., 2013; Kamat et al., 2013). Of note, similar effects have been observed for NO-donors currently tested as novel drug candidates in AD (Chegaev et al., 2015).

### Perivascular inflammation

Cerebral amyloid angiopathy is also associated with development of perivascular inflammation, microglial and astroglial activation, cytokines, and chemokines production. In addition, brain tissue hypoperfusion results in hypoxic alterations overlapping with Aβ-induced neurodegeneration. NO and H_2_S are well-known to exhibit anti-inflammatory effects, but dysregulated production of these gaseous transmitters at the sites of inflammation may promote degenerative changes. CBS producing H_2_S is localized to astroglial cell lineage and is up-regulated in reactive astrocytes (Kimura, 2015). Elevated expression of iNOS in brain endothelial cells is a known common feature of Alzheimer's type of vascular pathology associated with hypoxic injury (Sanchez et al., 2012; Figure 2). However, little is known about a putative role of H_2_S in perivascular inflammation in AD, despite this gaseous substance may exhibit neuroprotective effects due to anti-inflammatory activity (Giuliani et al., 2013). Similar effects have been demonstrated for CO (Cuadrado and Rojo, 2008).

### Cerebral hypoperfusion and endothelial dysfunction

Alzheimer's type of neurodegeneration starts from chronic cerebral hypoperfusion. It is interesting to note that metabolism of an allosteric activator of CBS—S-adenosyl-l-methionine (SAM)—is altered in chronic cerebral hypoperfusion (Wu et al., 2014). Thus, it is tempting to speculate that disturbances in SAM cycle would affect CBS activity leading to impaired H_2_S synthesis in the affected brain regions. Moreover, AD progression in humans is associated with decreased levels of SAM in the cerebrospinal fluid (Linnebank et al., 2010), while SAM has been proved to serve as cognitive-enhancing agent in AD animal model (Montgomery et al., 2014). Previously, such data have been recognized as evidence of altered methylation patterns seen in AD, but probably the summarized picture should include diminished activity of CBS in SAM-deficient brain.

Homocysteine is one of the most important risk factors of AD inducing endothelial dysfunction, angiopathy, and memory deficits. Toxic effects of homocysteine and the product of its spontaneous oxidation, homocysteic acid, are linked to activation of NMDA receptors, induction of intracellular calcium ion imbalance and oxidative stress (Boldyrev, 2009; Boldyrev et al., 2013). Homocysteine metabolism actually links NO and H_2_S pathways in AD (Figure 2). Both NO and H_2_S protect vascular and neuronal cells from homocysteine-induced injury (Dayal et al., 2014; Wei et al., 2014). Elevated plasma homocysteine level leads to reduction in nitric oxide bioavailability due to suppression of NOS activity (Lai and Kan, 2015). At the same time, homocysteine can be converted to l-cysteine and H_2_S due to activity of CBS and CSE, however, toxic concentrations of homocysteine suppress CBS and CSE activities in the brain along with increased expression of NMDA receptors in neuronal cells (Kamat et al., 2015a). These effects are linked to excitotoxicity and blood-brain barrier disruption. Thereby, activity of H_2_S- and NO-generating enzymes is decreased in AD, and homocysteine-mediated toxicity results in endothelial cell loss and progression of neurodegeneration (Figure 2). Recent findings suggest that exogenous H_2_S supply may normalize dysregulated expression of NMDA receptors, CBS, and CSE in renovascular diabetic remodeling (Kundu et al., 2015). H_2_S is able to restore decreased levels of eNOS, CD31, VE-cadherin, and endothelin-1 expression in brain endothelial cells subjected to the toxic action of homocysteine *in vitro* (Kamat et al., 2015b) and *in vivo* (Kamat et al., 2013), however whether or not this effect can be reproduced in cerebral amyloid angiopathy remains to be elucidated.

### Nitrosation and sulfhydration of vessel proteins in AD

Less attention has been paid to alternative molecular mechanisms of H_2_S functioning in the central nervous system. By analogy to NO which induces reversible nitrosation of target proteins, H_2_S may induce reversible protein modification (persulfidation, or alternatively S-sulfhydration; Gadalla and Snyder, 2010). Presumably, CSE (or other enzymes?) generates H_2_S to persulfidate the targets. Current evidence suggests that protein persulfidation adheres closely to the generally acknowledged paradigm for S-nitrosation by NO. Whereas, nitrosation appears to diminish cysteine reactivity, persulfidation seems to enhance it. Persulfidation may mediate various reported physiological actions of H_2_S, i.e., relaxation of blood vessels through the endothelial-derived relaxing factor activity of H_2_S involves opening of ATP–sensitive potassium channels (Yang et al., 2008), which are substrate for posttranslational modifications. Persulfidation and tyrosine nitration have been reported to occur at different subunits of K_ATP_channels, SUR2B, and Kir6.1, respectively, and pretreatment of the cells with H_2_S donor NaHS results in the suppression of NO-induced nitration (Kang et al., 2015). K_ATP_ channels protect endothelial cells (Chen et al., 2015), control release of NO and eicosanoids further acting on smooth vessel cells to produce vasorelaxation (Minamino and Hori, 2007). Besides that, Kir6.1 is a pore-forming subunit in astroglial plasma membrane K_ATP_ channels (Thomzig et al., 2001), which are involved in the intercellular communication within the neurovascular unit (Velasco et al., 2000; Sun and Hu, 2010). Thus, coordinated action of H_2_S and NO on K_ATP_ channels expressed in endothelial, astroglial and vascular smooth muscle cells may be an important regulatory mechanism of cerebral vasomotor activity impaired in AD.

Among the proteins modified by S-nitrosation, ryanodine receptors (RyR) are well-known targets, and NO-mediated S-nitrosation of RyRs mediates calcium release in neuronal cells (Kakizawa et al., 2013). In physiological conditions, RyRs are activated by cyclic adenosine diphosphate-ribose (cADP-ribose), a product of catalytic activity of NAD+-glycohydrolase/CD38 (Higashida et al., 2007). RyR2 and RyR3 are expressed in astrocytes, and RyR-dependent signaling has also been reported in vascular endothelium where three RyR isoforms have been identified. RyR3 are important for astrocytes migration (Matyash et al., 2002). RyR3 appears to be more broadly expressed, with predominance in neurons, and the three subtypes are expressed in large cerebral arteries as well as in the cerebral microcirculation (Dabertrand et al., 2013). Key role of RyR in Aβ production and learning and memory performances has been proposed (Oulès et al., 2012). In AD, there is a dysregulated expression of RyR, particularly in RyR2 splice variants (Bruno et al., 2012) and RyR3 (Liu et al., 2014a). Currently, there are no data on NO-mediated nitrosation or H_2_S-mediated sulfhudration of RyR in AD. However, the wide spectrum of S-nitrosated proteins seen in AD (Zahid et al., 2014; Zaręba-Kozioł et al., 2014) and very recent data on the inhibitory action of H_2_S-donor on RyR activity do not rule out this possibility (Tomasova et al., 2015).

### Mitochondrial dysfunction in endothelial cells

Brain endothelial cells have much higher density of mitochondria comparing to endothelial cells in other tissues (Oldendorf et al., 1977), and mitochondrial activity is extremely important for the regulation of endothelial cell metabolism (Salmina et al., 2015). Therefore, production of NO and H_2_S in or their action at mitochondria of endothelial cells should be considered in the context of AD pathogenesis. As expected, mitochondrial failure associated with energy shortage, calcium ion imbalance, and reactive oxygen species overproduction has been repeatedly confirmed in AD. These processes link mitochondrial dysfunction to neurodegeneration and neuroinflammation as well as to the blood-brain barrier dysfunction and impairment of cerebral endothelium repair (Sochocka et al., 2013).

Mitochondria represent the “cross-road” for the gaseous transmitters within the cells. Mitochondrial location was demonstrated for CSE, CBS (due to translocation in stressed cells), 3MST, and NOS. H_2_S inhibits mitochondrial production of reactive oxygen species, supports oxidative phosphorylation and prevents apoptosis (Guo et al., 2012; Szabo et al., 2014). Dose-dependent effects for H_2_S activity on mitochondrial function was demonstrated: low concentrations (below 1 μM) stimulate mitochondrial respiration, while high concentrations (higher than 3 μM) inhibit mitochondrial respiration. Such effects of H_2_S require adequate levels of Krebs cycle activity, therefore a co-ordinatory role of intramitochondrially produced H_2_S in the connection of citric acid cycle and oxidative phosphorylation has been proposed (Módis et al., 2013). NO is involved in the regulation of multiple aspects of mitochondrial functioning due to its ability to bind heme iron centers, to nitrosylate proteins (i.e., complex I) to inhibit electron flux, and to take part in reactive oxygen species formation (Stefano and Kream, 2015). Activation of mitochondrial K_ATP_ channels in cerebral endothelial cells leads to NOS activation, NO production, and vasodilation (Katakam et al., 2013) whereas insulin resistance-associated impairment of K_ATP_ channels alters above-mentioned mechanism (Katakam et al., 2009).

In AD, aberrant K_ATP_ channels activity and endothelium-mediated vasodilation was reported many years ago (Chi et al., 1999). At present, these data could be interpreted as an indirect effect of NO or H_2_S insufficiency resulting in mitochondrial impairment. Of note, CO is also known to induce uncoupling of mitochondrial respiration dependent on the activation of mito BK_Ca_ channels and inhibition of glycolysis independent of mito BK_Ca_ channels (Kaczara et al., 2015). Thus, all three gaseous transmitters demonstrate different behavioral patterns in mitochondria: stimulation or inhibition of mitochondrial respiration by H_2_S, uncoupling of mitochondrial respiration by CO, and inhibition of respiration by NO.

Mitochondrial biogenesis in endothelial cells is one of the requirements for effective angiogenesis, and is usually associated with the suppression of glycolysis. NO stimulates mitochondrial biogenesis in many cell types (Nisoli et al., 2004; Miller et al., 2013), H_2_S maintains mitochondrial DNA copy number that is important for mitochondrial biogenesis (Li and Yang, 2015), thereby exogenous H_2_S supports mitochondrial biogenesis in brain cells subjected to hypoxia/ischemia (Pan et al., 2014). Thus, suppression of mitochondrial biogenesis seen in AD (Rice et al., 2014; Burté et al., 2015) might be, at least partially, caused by reduced levels of endogenous NO and H_2_S. On the other hand, accumulation of S-nitrosocysteines due to NO-mediated nitrosation of mitochondrial proteins may have direct toxic effect especially under the conditions of oxidative stress: NAD^+^ depletion, ATP deficit, mitophagy induction in endothelial cells similarly to previously obtained data (Diers et al., 2013). However, it should also be considered that H_2_S may efficiently suppress homocysteine-induced mitophagy in cerebral endothelial cells (Kamat et al., 2015b), thereby acting also as a functional antagonist of NO.

### Aberrant angiogenesis and neurogenesis in AD

Compromised angiogenesis takes place in AD, and plays a role in the progression of neurodegeneration. Aβ promotes neoangiogenesis and hypervascularity (Biron et al., 2011) that is supported by the elevated levels of proangiogenic factors—angiogenin and tissue inhibitor of matrix metalloproteinase-4—in the plasma of patients with AD (Qin et al., 2015). However, the present data are controversial since other authors found decreased serum levels of angiogenin in AD (Kim and Kim do, 2012). In both studies, there was a correlation between the levels of angiogenin and the severity of cognitive impairment, probably due to assessment of disease manifestations at different phases of progression. Other studies found that angiogenin stimulates NO synthesis and release from endothelial cells (Trouillon et al., 2010), whereas H_2_S increases angiogenin expression in endothelial cells (Geng et al., 2015). Thus, it is tempting to speculate that this machinery—[H_2_S → angiogenin → NO]—is dysregulated in AD, thereby contributing to abnormal angiogenesis.

H_2_S and NO themselves serve as potent angiogenic molecules acting individually or in the combination with other angiogenic factors to promote endothelial progenitor cells migration, proliferation (Lee et al., 2009; Kimura, 2015; Lu et al., 2015; Figure 1). H_2_S activates K_ATP_ channels that possess angiogenic properties being involved into the cell response to the action of VEGF, and contribute to NO release from endothelial cells (Umaru et al., 2015). The Kir6.1 subunit of K_ATP_ channels, which is target for NO-induced nitration (Kang et al., 2015), is required for the angiogenic activity of VEGF (Umaru et al., 2015). Thus, the possibility arises that decreased and/or imbalanced production of H_2_S and NO observed in AD does diminish the angiogenic potential of factors acting through K_ATP_ channels.

Angiogenesis is important not only for reparative processes in adult brain, but also for supporting neuroplasticity. Multiregional dysregulation of neurogenesis, impaired neuronal migration and maturation are common in AD (Hamilton et al., 2010). The vascular microenvironment within the neurogenic niche is integrated by signaling molecules secreted from endothelial cells in the brain vasculature or by direct contact with these cells (Goldberg and Hirschi, 2009). Endothelial-secreted factors from the brain vasculature regulate proliferation, survival/self-renewal, differentiation, and migration of neural stem/progenitor cells within the neurogenic niches. There are many paracrine effectors of the brain vasculature on neurogenesis, such as VEGF, EGF, bFGF, Notch ligands. It is known that H_2_S directly activates VEGF receptor 2 (VEGFR2), thus providing pro-angiogenic effect (Tao et al., 2013).

Recent finding suggest that constituent release of VEGF from neural stem/progenitor cells highly expressing VEGFR2 in the neurogenic niches supports their functional activity (Ara et al., 2010; Kirby et al., 2015). Therefore, local production of H_2_S could potentiate these paracrine and autocrine regulatory processes supporting neurogenesis and angiogenesis. H_2_S and l-cysteine are also known to promote proliferation of neural stem cells (Wang et al., 2013a; Liu et al., 2014b). RyR2 expressed in neural stem/progenitors embryonic cells take part in neurogenesis (Yu et al., 2008). At the same time, endogenous production of NO downstream of RyR activation is required for the positive regulation of proliferation of hippocampal neural progenitor cells derived from embryonic mice (Yoneyama et al., 2011). However, impairment of neural stem cells proliferation due to NO-mediated nitration of the EGF receptor and suppression signaling through the ERK/MAPK pathway was reported (Carreira et al., 2014). Together, targeting H_2_S and NO as putative local regulators of neurogenesis within the neurogenic niches may represent a novel approach for restoration of brain cell proliferation and differentiation in AD (Figure 2).

## Conclusion

Being produced by the cells of the neurovascular unit, H_2_S, and NO act mainly as functionally additive molecules contributing to the regulation of the local blood flow, maintenance of endothelial integrity, and controlling intercellular communications. Synergistic action of physiological levels of NO and H_2_S is apparent in their neuroprotective, angiogenesis promoting activity as well as in the gliovascular control providing adequate blood supply to the active brain zones. Therefore, amyloid-induced suppression of NO and H_2_S production in endothelial and astroglial cells results in impairment of endothelial function and cerebral microcirculation. Also, acting at the same direction, NO, and H_2_S suppress Aβ production and deposition in cerebral microvessels. However, antagonism in the action of two gaseous transmitters is evident in their ability to modify proteins: NO-induced nitrosation usually suppress the activity of target proteins, whereas H_2_S-induced sulfhydration leads to activation of target proteins. Contrary to H_2_S that maintain mitochondrial DNA copy number, NO stimulates mitophagy in endothelial cells. However, it should be noted that data obtained with NO and H_2_S in complex biological systems should be considered very carefully because of dose-dependent action of both the gaseous mediators on cell metabolism and functioning: the final outcome could be diametrically opposite depending on the actual concentration. That is why our current understandings on the role of NO and H_2_S in (patho)physiological conditions are very far from the resultant conclusions.

It is clear that Alzheimer's type of neurodegeneration dramatically affects H_2_S and NO synthesis and their interactions resulting in dysregulated vasomotor function, brain tissue hypoperfusion and hypoxia, development of perivascular inflammation, promotion of Aβ deposition, and impairment of neurogenesis/angiogenesis (Figure 2). Better understanding of key cellular, molecular, and pathobiochemical mechanisms of H_2_S and NO action will provide new directions for the development of high-performance technologies for neural regeneration and neuroprotection with potential impact in clinical medicine.

### Conflict of interest statement

The authors declare that the research was conducted in the absence of any commercial or financial relationships that could be construed as a potential conflict of interest.
